# The impact of loneliness and social isolation on health state utility values: a systematic literature review

**DOI:** 10.1007/s11136-021-03063-1

**Published:** 2022-01-24

**Authors:** Ishani K. Majmudar, Cathrine Mihalopoulos, Bianca Brijnath, Michelle H. Lim, Natasha Yvonne Hall, Lidia Engel

**Affiliations:** 1 Faculty of Health, School of Health and Social Development, Institute for Health Transformation, Deakin Health Economics, Deakin University, Geelong, VIC Australia; 2 Social Gerontology, National Ageing Research Institute (NARI), Parkville, Australia; 3 Centre for Mental Health, Swinburne University of Technology, Hawthorn, Australia

**Keywords:** Loneliness, Social isolation, Health state utility values, Economic burden, Health burden

## Abstract

**Background:**

Loneliness and social isolation are recognised as social problems and denote a significant health burden. The aim of this study was to conduct a systematic literature review to explore the health state utility values (HSUVs) associated with loneliness and/or social isolation.

**Method:**

Peer-reviewed journals published in English language that reported both HSUVs along with loneliness and/or social isolation scores were identified through five databases. No restrictions were made relating to the population, study design or utility estimation method used.

**Results:**

In total, 19 papers were included; 12 included a measure of loneliness, four studies included a measure of social isolation and three studies considered both loneliness and social isolation. All studies focused on individuals with pre-existing health conditions—where the EQ-5D-3L instrument was most frequently used to assess HSUVs. HSUVs ranged from 0.5 to 0.95 in those who reported not being lonely, 0.42 to 0.97 in those who experienced some level of loneliness, 0.3 to 0.87 in those who were socially isolated and 0.63 to 0.94 in those who were not socially isolated.

**Conclusion:**

There was significant variation in HSUVs complicated by the presence of co-morbidities, population heterogeneity, variations in methods used to derive utility scores and differences in the measurement of loneliness and/or social isolation. Nevertheless, the lower HSUVs observed should be considered to significantly impact quality of life, though we also note the need for further research to explore the unique impact of loneliness and social isolation on HSUVs that can be used in the future economic evaluations.

**Supplementary Information:**

The online version contains supplementary material available at 10.1007/s11136-021-03063-1.

## Background

Loneliness and social isolation represent global public health concerns [1, 2]. While the terms are often used interchangeably, they are not synonymous; social isolation is an *objective* state and increased social isolation can be quantified by the reduced size of social networks and lack of social contact. Loneliness, also referred to as ‘perceived social isolation’, on the other hand, is a *subjective* experience, which occurs when a person feels a discrepancy between desired and actual social relationships [3, 4]. Thus, social isolation is different from loneliness as one can have social connections and still feel lonely, or can be alone and may not feel lonely [5].

While every individual will experience loneliness at some point in their life, some age groups are more prone to loneliness, for example, late adolescence or older people [6]. Critical transitions during these life stages, from adolescence to adulthood, as well as decreasing economic and social resources, limitations in mobility and loss of spouse and relatives that are common in later life, are thought to explain why these age groups experience more loneliness and social isolation [6, 7]. Previous literature has demonstrated a link between loneliness and social isolation with increased risk of developing cardiovascular diseases [8], cognitive deterioration [9], increased blood pressure [10], infectious illnesses [11] and early mortality [12]. Loneliness and social isolation are also associated with increased risk of dementia [13], depression and suicide [14]. Moreover, it has been argued that the health impact of loneliness and social isolation can be worse than risk factors such as smoking or obesity [15]. It has also been found that older adults who experience “extreme loneliness” have a greater chance of premature death [16].

Ongoing strategies to tackle loneliness and social isolation through interventions have been developed over years [17–19]. Many of these interventions targeted older adults and reported some level of success in reducing loneliness and/or social isolation with factors such as adaptability, community development approach and productive engagement being associated with the most effective interventions [20]. However, whether such interventions provide ‘good value for money’ has largely remained unanswered. Given the scarcity of healthcare resources, it is important to identify not only effective interventions and programmes but also cost-effective strategies [19]. The most common type of economic evaluation is cost-utility analysis (CUA), which expresses results in terms of cost per quality-adjusted life year (QALY) gained [21]. To generate QALYs, length of life is adjusted by levels of health-related quality of life (HRQoL) using a single value known as health state utility value (HSUV). HSUV is measured on a scale of 0–1, where 0 is equivalent to being dead and 1 is considered as perfect health [21]. While HSUV can be obtained using direct techniques (i.e. time trade-off, standard gamble or visual analogue scale), these weights are commonly derived indirectly through the use of questionnaires, referred to as multi-attribute utility instruments (MAUIs) or preference-based HRQoL instruments. These instruments consist of a descriptive system, where a set of items (or questions) elicits responses to the main dimensions of HRQoL measured by the questionnaire. A second component of MAUIs is the valuation system. MAUIs have utility formula attached to each response on an item/domain, which returns a weight anchored between 0 and 1 for each set of responses. This formula reflects societal preferences that indicate the relative importance of each item and in turn each HRQoL domain to derive a single HSUV [21]. These values are used to compare the preferences of the general population for different health states across various diseases [22]. Therefore, MAUIs allow an intervention targeted to reduce loneliness to be compared with any other health condition. HSUVs and QALYs are commonly used outcome measures across all areas of economic evaluation and Health technology assessment agencies across the world.

The EQ-5D(-3L or -5L) [23], Health Utilities Index Mark 2 or Mark 3 (HUI2 and HUI3), Short-Form-6 Dimension (SF-6D)—derived from the SF12 or 36, quality of well-being (QWB) scale, the assessment of quality of life (AQoL) suite of instruments and 15 Dimension (15D) are the most popular MAUIs [24]. They differ in their conceptualisation, content, length and methods used for converting health state descriptions into utilities. No single instrument is considered a gold standard for a certain health state, although some countries have expressed a preference for a particular MAUI in their national guidelines (e.g. the United Kingdom prefers the EQ-5D-3L) [22]. The EQ-5D, HUI, QWB and 15D have items predominantly relating to physical health. The SF-6D has an equal number of items covering both physical and psychological domains, while the AQoL-8D has been developed to capture particularly psychosocial domains of HRQoL, including social functioning [24]. Using a suitable utility method and instrument can affect the estimated HSUVs and in turn influence cost-effectiveness results as different MAUIs produce different HSUVs even in the same people [25]. This is largely because the different MAUIs measure different HRQoL domains.

Economic evaluations can be conducted alongside clinical trials or via modelling techniques. Appropriate HSUVs are assigned to health states defined within a model for economic evaluation that individuals experience over time through their treatment pathway. HSUVs associated with these health states thus provide important inputs to conduct cost-effectiveness analysis. As it is practically not feasible for health economists to elicit individual HSUVs directly or indirectly for every study, reviews of HSUVs for use in individuals across health states can provide a readily available source of HSUVs that can be used in an modelled economic evaluation [25–27]. These reviews provide results that improve robustness, transparency and rigour of the economic model, allowing appropriate and systematic selection of model parameters as well as greater understanding of the ‘burden of disease’ for a particular health state. Although, several studies have previously conducted reviews to explore the health and economic burden of different health conditions [26, 28, 29], to the best of our knowledge, no published review has examined HSUVS by the presence/absence or the degree of loneliness and social isolation.

We aimed to systematically review the literature to assess the availability of HSUVs associated with loneliness and/or social isolation across all age groups, with the intention that the findings can inform future model-based economic evaluations as well as provide an indication of the burden potentially associated with loneliness and social isolation. This review will help demonstrate which measurement techniques were used to assess HSUVs, to inform future trial-based economic evaluation and provide information on the corresponding value of loneliness and/ or social isolation scores.

## Methods

A comprehensive systematic literature review was conducted according to the preferred reporting items for systematic reviews and meta-analysis (PRISMA) [30]. This review protocol was registered on the Prospero database [CRD42021243375].

### Study selection

The search was conducted in November 2019 and updated in April 2021. The five databases searched included Medline Complete, PsycINFO, Embase, CINAHL and EconLit. A keyword search was performed using the terms related to concepts of loneliness, social isolation, measures of loneliness and measures of HSUVs. Search terms for each concept were combined along with Medical Subject Headings (MeSH) terms (Supplementary 1). Initial title abstract screening was conducted by two reviewers  IM and NH using the Rayyan QCRI Tool [31]. A third author LE resolved disagreements. Full-text screening of the agreed articles from the first step was conducted by two authors with any discrepancies solved through discussion with the third author. Third, a backward citation search, which involved screening the titles and abstracts of the reference list of the included studies, was conducted using Scopus. All types of studies (observational and experimental designs) that have used direct elicitation methods or indirect methods to measure HSUVs and have corresponding loneliness/social isolation scores were included. The inclusion and exclusion criteria are stated in Table 1.

**Table 1:** Inclusion criteria and exclusion criteria

	Inclusion criteria	Exclusion criteria
Population	All populations and age groups	
Outcome of interest	Loneliness and/or social isolation	
Type of study	Quantitative studies (cross-sectional, longitudinal, cohort, etc.), reported the scores of utility weights relating to loneliness or social isolation	Qualitative studies
Utility instrument	Both direct elicitation methods and indirect utility valuation methods	
Country	All countries	
Publication type	Published in peer-reviewed journals	Protocol papers, conference abstracts, reviews, expert opinion and editorials
Year of Publication	No restriction	
Language	English Language	Other Language

### Data extraction

Data extraction was conducted by the lead author and checked by another author. To guide data extraction, pre-designed tables were used. Adjustments to the tables were made to accommodate the information provided by the included articles.

The data extracted included the following:

(i) Descriptive data about lead author, publication year, country, study design and sample size;
(ii) Demographic data about target population, including presence of health conditions;
(iii) *Utility valuation data*: Utility score methods with reported mean utility value;
(iv) *Loneliness and social isolation measure data*: Measure of reporting loneliness and/or social isolation with the corresponding mean score.

We aimed to include all studies which reported a HSUV score alongside a loneliness/social isolation score, not limiting to studies that only included study participants who experienced loneliness and/or social isolation. HSUV scores were extracted either for the entire study sample along with a corresponding score from the loneliness measure and/or social isolation measure, or where available, by levels of loneliness and social isolation using instrument-specific cut-off points. Where the allocation of HSUVs by levels of loneliness and/or social isolation was not provided by authors, a categorisation of scores was made by the lead author based on the reported values and the cut-off points of the loneliness/social isolation measures used in the study by Zhu et al. [32]. Baselines scores were considered in studies which reported HSUVs at different time points.

## Results

**Fig. 1:**
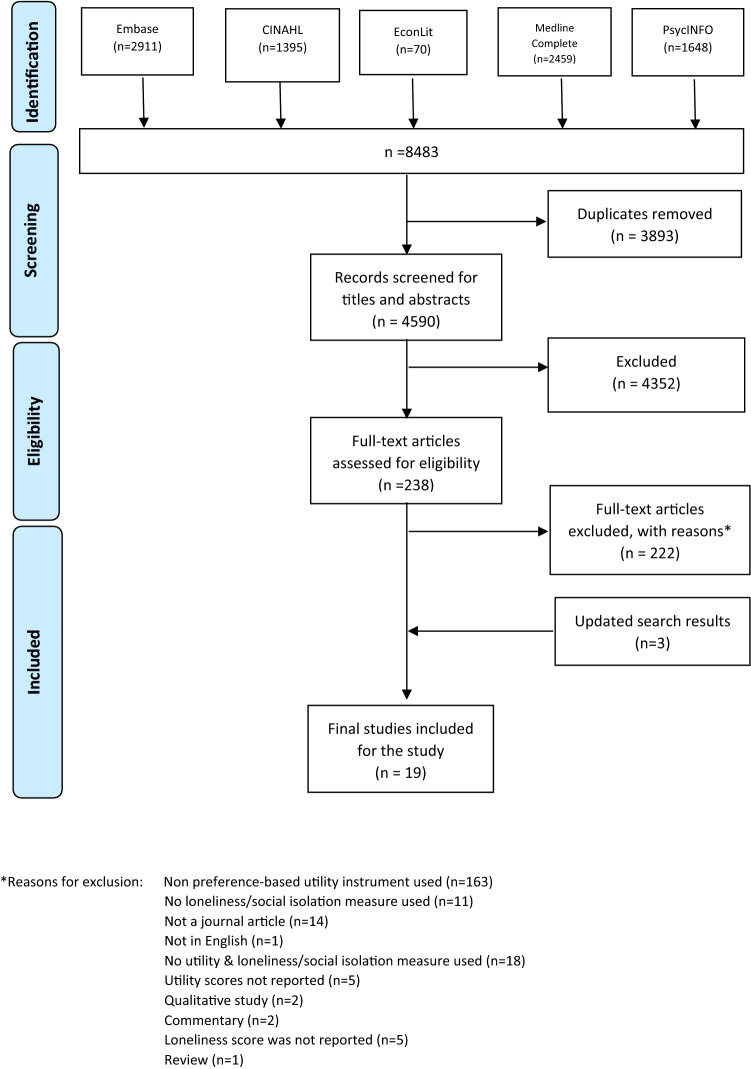
PRISMA Diagram

After deduplication, our search yielded 4590 unique references. After screening titles and abstracts, we reviewed 238 full texts, of which 16 studies met inclusion criteria. An updated search was undertaken, which further identified three studies. The PRISMA flow diagram, presented in Fig. 1, shows the selection process with reasons of exclusion.

### Study characteristics

There has been an increasing interest in the research area of loneliness and health outcomes over the last decade, as most of the included studies were published after 2010. Sample size varied from 20 to 2713 individuals in the reviewed studies. Included articles consisted of studies from Australia (*n* = 4) [33–36], UK (*n* = 3) [37–39], Singapore (*n* = 2) [40, 41], Netherlands (*n* = 2) [42, 43], Canada and USA (*n* = 2) [44, 45], with one study from the Republic of Korea [46], China [32], Spain [47], Finland [48], Sweden [49] and Malaysia [50]. Thirteen studies included an older population [32, 34, 36, 38–40, 42, 44, 46–50] five studies included general adults [35, 37, 41, 43, 45] and one study focused solely on young adults aged 18–25 years [33]. There were nine cross-sectional studies [32, 38, 41, 44–47, 49, 50], eight interventional studies [34, 36, 37, 39, 40, 42, 43, 48], one observational study [33] and one quasi-experimental study [35]. Ten studies included community-dwelling participants [32, 37–42, 44, 48, 49], two studies focused on institutionalised vs non-institutionalised older adults, while the remaining studies did not clearly specify the living situation of study participants [45, 47, 50]. Two studies recruited participants specifically experiencing loneliness [43, 48] while most studies included participants with various other health conditions, such as sight loss [37], hearing loss [36], serious mental illness [33, 50], depression [46], skin disease [41], HIV [45] and multiple co-morbidities [34, 35, 40, 42, 44, 47, 48].

HSUVs were reported in twelve studies alongside a measurement of loneliness, four studies alongside a measure of social isolation and three studies considered both. There were inconsistencies in the way loneliness and social isolation were reported and very few studies categorised the study sample into degrees of loneliness and/or social isolation [32, 38]. Table 2 describes the overview of instruments used. Further characteristics of studies that reported HSUVs by level of loneliness and social isolation are presented in Table 3 and 4, respectively.

**Table 2:** Overview of instruments used

Utility		Loneliness		Social isolation	
Measure	#	Measure	#	Measure	#
EQ-5D (-3L/-5L)	12 [32, 34, 37, 39–43, 45–47, 49]	UCLA LS 20 item	3 [32, 37, 46]	Lubben Social Network Scale-revised	1 [36]
AQoL-8D	3 [33, 35, 50]	Six-item De Jong Gierveld Loneliness Scale	1 [47]	The Lubben Social Network Scale- 6 item	2 [40, 41]
HUI2	1 [44]	De Jong Gierveld Loneliness Scale 11-item scale	4 [36, 39, 42, 43]	Friendship Scale	2 [34, 50]
HUI3	1 [36]	R-UCLA LS 3 item	2 [35, 41]	Social inclusion	1 [33]
15D	1 [48]	R-UCLA LS 20 item	1 [33]	Social health battery	1 [38]
EQ-5D + SF-6D	1 [38]	Single question	5 [35, 44, 45, 48, 49]

**Table 3:** Study Characteristics and reported utility weights by level of loneliness from extracted studies

Author, Country	Population characteristics	Study design (Sample size)	Reported health conditions	Utility instrument (tariff used)	Loneliness measure	Overall population mean loneliness score (SD) mean utility (U) score (SD)		Mean utility score (U) for those ‘not lonely’, ‘lonely’ and by levels of loneliness*				
								Not lonely	Lonely	Low loneliness	Moderate loneliness	Moderately High loneliness
Zhu et al., China [32]	≥ 60 years, mean age (SD): 71 (7.73) Community-dwelling women: 51%, Married: 64%, Empty nester: 46.6%	Cross-sectional design (*n* = 732)	Chronic disease 76.4%	EQ-5D-3L (Chinese general population)	20-item UCLA LS^a^	40.73 (8.73) U: 0.93 (0.12)				*20–34:* U: 0.97	*35–49:* U: 0.93	*50–64:*^b^ U: 0.86
Ko et al., South Korea [46]	≥ 60 years, mean age (SD): 77 (5.87) Women: 78% All Living alone Widowed: 76%	Cross-sectional design (*n* = 1023)	Mild to severe cognitive impairment: 57.2% Moderate depression 26%	EQ-5D-3L (UK general population)	20-item UCLA LS^a^	41.54 (13.12) U: 0.81 (0.18)					*35–49*^c^ 41.54 U: 0.81	
Rodriguez-Blazquez et al., Spain [47]	≥ 60 years older adults *Non-institutionalised group* mean age (SD): 72 (8.16) Without partner:43% Women: 58% *Institutionalised group* mean age (SD: 81 (7.06) Without partner:81% Women: 65%	Cross-sectional design (*n* = 468)	Mean number of medical conditions (SD) *Non-institutionalised* 3.57 (2.62) *Institutionalised* 6.50 (2.79)	EQ-5D-3L (not stated)	6-item De Jong Gierveld loneliness Scale ^d^	*Non- institutionalised* 2.02 (1.81) U: 0.81 (0.26)	*Institutionalised* 2.54 (1.58) U: 0.57 (0.36)		*2–6:*^d^ 2.02 U: 0.81 2.54 U: 0.57			
Liira et al., Finland [48] based on Pitkala et al. [67]	≥ 75 years: mean age (SD): 80 (4) Home dwelling Women: 73% Widowed: 68%	RCT for psychosocial group intervention to explore HRQoL (*n* = 235)	Having subjective feelings of loneliness and numerous health conditions	15D (not stated)	Study specific single question ^f^	NR U: 0.78 (0.12) ^g^			*Yes:* *U:* 0.78			
Taube et al., Sweden [49]	≥ 65 years, mean age (SD): 82 (6.4) Women: 67% *All Home dwelling* *Not lonely sample* (*n* = 61) mean age (SD): 81 (6.5) Windowed 21.3% *Lonely sample* (n = 61) mean age (SD): 82 (6.2) Windowed 67.4%	Cross-sectional design (*n* = 153) *Not Lonely* (n = 61) *Lonely* (*n* = 92)	Dependency in daily activities, repeated contacts with the health care services *Not lonely*: No of health complaints: 9.8 (4.7) *Lonely*: No of health complaints: 12.1 (4.6)	EQ-5D-3L (UK general population)	Study specific single question (Yes/No) ^h^	NR U: 0.59 (0.27)		*No:* U: 0.63	*Yes:* U: 0.56			
Van Houwelingen et al., Netherland [42]	≥ 75 years median age 82 *All Home dwelling* Living alone 55 Widowed 53% Women: 68%	RCT to assess efficacy of a simple structural monitoring system to detect deterioration in the functional, somatic, mental or social health of individuals (*n* = 2713)	No. of chronic diseases^i^ median (IQR): 4 (3–6)	EQ-5D-3L (UK general population)	11-item De Jong Gierveld Loneliness Scale ^j^	Problems *Functional*:^k^ 3 U: 0.65 *Somatic*: 2 U: 0.69 *Mental*: 3 U: 0.69 *Social* 4 U: 0.72	No problems *Functional:* 2 U: 0.81 *Somatic*: 1 U: 0.84 *Mental*: 1 U: 0.81 *Social* 1 U: 0.81	*0–2* ^j^ 2 U: 0.81 2 U: 0.69 1 U: 0.84 1 U: 0.81 1 U: 0.81			*3–8*^j^ 3 U: 0.65 3 U: 0.69 4 U:0.72	
Gardner et al., Australia [33]	18–25 years old: Mean age (SD): 21 (2.21) Women: 48% Living with family: 72%	Observational study (*n* = 159)	Serious mental illness^e^	AQoL-8D (Australian general population)	20-item R-UCLA LS^c^	52.48 (12.94) U: 0.42 (0.21)						50–64^c^: 52.48 U: 0.42
Mountain et al., UK [39]	≥ 65 years mean age (SD): 72 (65.92) Community living Women: 68% Normal cognition Living alone 55%	RCT involving weekly group sessions Intervention (*n* = 145) Control (*n* = 143)	No reported health conditions	EQ-5D-3L (UK general population)	11-item De Jong Gierveld Loneliness Scale ^j^	*Control* Baseline: 4.6 (3.6) U: 0.77 (0.24) 6 months 4.1 (3.4) U: 0.76 (0.23) 24 months: 4.8 (3.6) U: 0.71 (0.28)	*Intervention* Baseline: 4.1 (3.5) U: 0.73 (0.25) 6 months: 3.5 (3.2) U: 0.71 (0.25) 24 months 3.7 (3.4) U: 0.73(0.24)				*3–8* ^j^ 4.1 m U: 0.73 4.6 U: 0.77 3.5 U: 0.71 4.1 U: 0.76 3.7 U: 0.73 4.8 U: 0.71	
Maxwell et al., Canada and USA [44]	≥ 65 years mean age (SD): 81 (8.4) Home care clients; Women: 72% Widowed 54% Not lonely 77% Self-rated health: Good/Excellent 69%	Cross-sectional design (*n* = 514)	More than 3 chronic diseases^#^: 75.3%	HUI2 (Canadian adult population)	Self-reported loneliness status (Yes/No)	NR U: 0.49 (0.18)		*No:* U: 0.50	*Yes:* U: 0.46			
Action et al., UK [37]	Mean age (SD): 75 (16.21) *All Home dwelling*	RCT to examine the effect of a home visit–based visual rehabilitation intervention (*n* = 67)	Sight loss causing difficulties in carrying out daily tasks	EQ-5D-5L (UK general population)	20-item UCLA LS^n^	*Control* Baseline: 10.47 (13.81) U: 0.60 (0.3) 6-months: 12.81 (14.71) U: 0.58 (0.29)	*Intervention:* Baseline: 13.14 (13.34) U: 0.51 (0.31) 6-months: 14.51 (15.19) U: 0.52 (0.34)					
Packer et al., Australia [35]	*Living with diabetes* mean age (SD): 60 (11.0) Women: 48% Number of health conditions^k^: 2 (1.1) *Living with any chronic condition* mean age (SD): 70 (10.5) Women: 66% number of health conditions: 2.6 (1.3)	Quasi-ongoexperimental design to investigate the impact of generic and diabetes-specific self-management programmes (*n* = 485)	*Living with diabetes* (*n* = 222) *living with any chronic condition (n* = *236)*	AQoL-8D (Australian general population)	R-UCLA LS 3 item^o^ Single question (SQ) with four responses^p^	*Living with diabetes*: R-UCLA: 4.02 (1.6) U: 0.73 (0.2) *Living with any chronic condition* R-UCLA: 4.64 (1.7) U: 0.48 (0.2) *Living with diabetes*: SQ: 3.24 (0.7) U: 0.48 (0.2) *Living with any chronic condition* *SQ:* 3.49 (0.7) U: 0.73 (0.2)						
Sarant et al., Australia [36]	Mean age (SD) 72 (6.8)	Interventional study investigating the impact of cochlear implants (*n* = 20)	With severe–profound hearing loss [49]	HUI3 (Not stated)	11-item De Jong Gierveld Loneliness Scale ^j^	Baseline: 2.64 U:0.56 18-months: 1.39 U:0.67		*0–2* 2.64 U:0.56 1.39 U:0.67				
Weiss et al., Netherlands [43]	Median age: 60 years; IQR (48.3, 68.0) 77% living alone Low socioeconomic status	RCT to examine the effect of the positive psychology intervention ‘Happiness Route’ [HR] (*n* = 58) compared to ‘Customised care’ [CC] (*n* = 50)	Severely lonely High level of comorbidity (median health conditions = 3)	EQ-5D (Dutch population)	11-item De Jong Gierveld Loneliness Scale ^j^	Control Baseline 9.41 (0.29) U: 0.46 (0.05) 3 months 9.43 (0.39) U: 0.54 (0.06) 9 months 8.85 (0.52) U: 0.5 (0.06)	Intervention Baseline 9.41 (0.29) U: 0.46 (0.05) 3 months 9.43 (0.39) U: 0.54 (0.06) 9 months 8.85 (0.52) U: 0.5 (0.06)					*8–11* ^j^ 9.41^ m^ U: 0.46 9.43 U: 0.54 8.85 U: 0.5 9.41 U: 0.46 9.43 U: 0.54 8.85 U: 0.5
Marianne Harris et al., Canada [45]	≥ 35 Mean age: 53 years (8.3)	Cross-sectional study (*n* = 856)	Diagnosed with HIV for atleast a year	ED-5D-5L (Not stated)	Study specific single question^f^	NR U: 0.70 (0.19)^g^		*No:* U:0.88	*Yes* U:0.70			
Weng Yew et al., Singapore [41]	Mean age: 54.3 years (16.8) Women: 56.2% Community-dwelling adults	Cross-sectional study (*n* = 1510)	Skin diseases	EQ-5D-5L	R-UCLA LS 3 item^o^	*Having a skin disease* 3.5 (1.2) U: 0.89 (0.18) *Not having a skin disease* 3.3 (0.8) U: 0.95 (0.12)		*3–5* 3.5 U: 0.89 3.3 U: 0.95				

^a^UCLA LS 20-item scores range from 20 to 80, with greater scores defining greater degrees of loneliness. Scores range from 20 to 34 depict low loneliness, 35–49 moderate loneliness, 50–64 moderately high loneliness, 65–80 high loneliness

^b^A significant difference was found for the utility scores for groups with different degrees of loneliness (*p* < 0.001)

^c^Categorised by lead author into reported levels of loneliness based on UCLA LS 20 scoring cut-offs provided in Zhu et al. [32]. Scores range from 20–34 low loneliness, 35–49 moderate loneliness, 50–64 moderately high loneliness, 65–80 high loneliness

^d^The 6-item De Jong Gierveld scale ranges from 0 to 6, where scores ranging from 0 to 1 denote a state of not being lonely and scores 2–6 denote a state of being lonely

^e^Serious mental illness included depressive disorders, anxiety disorders, schizophrenia, personality disorder, trauma-related disorder, bipolar related disorder, eating disorder, obsessive–compulsive and related disorders, substance-related and addictive disorders

^f^Single question with three responses: “Do you suffer from loneliness” (seldom or never, sometimes, often or always)

^g^Utility score was provided for only those who reported to be lonely

^h^Single-item question with yes/no response: ‘Looking back over the last year, which response alternative corresponds best for you?’ (no = 0; yes = 1)

^i^Including self-reported diabetes, heart failure, malignancy, COPD, incontinence, arthritis, osteoporosis, dizziness, LUTS, depression, anxiety, dementia, vision, deafness, fracture, stroke/TIA, myocardial infarction

^j^De Jong Gierveld Loneliness Scale 11-item scale score range 0–11 scale with higher scores indicating more loneliness, categorised as 0–2 not lonely, 3–8 moderately lonely, 9–11 strongly lonely

^k^The paper examined loneliness and quality of life by four health indicators (functional, somatic, mental and social); as such six values are reported here, which were categorised by levels of loneliness

^l^Reported as Median [IQR]

^m^This RCT collected loneliness and utility data across 3 measurement points (baseline, 6-months, 24-months) for the intervention and the control group

^n^Original 20-item UCLA loneliness scale ranging from 0 to 60; no established cut-off points

^o^Scores of 3–5 denote “not being lonely” and scores 6–9 indicate “being lonely”

^p^Single question with four responses: always feel lonely (1); often feel lonely (2), sometimes feel lonely (3),never feel lonely (4)

^*^Utility scores were obtained from the overall population and by presence/absence and level of loneliness based on categorisation reported by authors or using established cut-off points. Since the 20-item UCLA scale does not have any established cut-offs, the scores were categorised as per the cut-offs reported by Zhu et al. [32]

**Table 4:** Study characteristics and reported utility weights by level of social isolation from extracted studies

Author, Country	Population characteristics	Study design (sample size)	Reported health conditions	Utility instrument (tariff used)	Social isolation measure	Overall population Overall social isolation score (SD) Mean utility score (SD)		Mean Utility scores (U) for those ‘not socially isolated’, ‘socially isolated’ and by levels of social isolation
Yap et al., France [40]	≥ 65 years mean age (SD): 75 (6.4) living in the community Living alone 33% Women: 94%	RCT to explore the effects of Rhythm-centred music making on HRQoL (*n* = 54)	More than 3 co-morbidities: 80%	EQ-5D-5L (Singapore general population)	The Lubben Social Network Scale 6-item^a^	*Intervention* T1: 18.50 (7.5,23)^b^ U:0.81 (0.67, 0.94) T2: 12.50 (8.5,18.5) U:0.94 (0.72, 1.00) T3: 12.00 (8,16) U:0.87 (0.69, 0.88)	*Control* T1: 10.00 (4,14) U:0.63 (0.28, 0.74) T2: 12.00 (6, 14) U:0.60 (0.35, 0.86) T3: 11 (6, 17) U:0.63 (0.39, 0.74)	*Not socially isolated (greater than 12)* 18.50, U:0.81 12.50, U:0.94 *Socially isolated (less than 12)* 12.00, U:0.87 11.00, U:0.63 12.00, U:0.60 11.00, U:0.63
Weng Yew et al., Singapore [41]	Mean age: 54.3 years (16.8) Women: 56.2% Community-dwelling adults	Cross-sectional study (*n* = 1510)	Skin diseases	EQ-5D-5L	The Lubben Social Network Scale 6-item^a^	*Having a skin disease* 15.9 (6.4) U: 0.89 (0.18) *Not having a skin disease* 16.6 (5.9) U: 0.95 (0.12)		*Not socially isolated (greater than 12)* 15.9, U:0.89 16.6, U:5.9
Nikmat et al., Malaysia [50]	Patients aged 60–89 years mean age (SD) 69 (6.97) *living in community* single/separated 42%; healthy 74% *nursing homes* mean age (SD): 72 (7.66) single/separated: 87%; healthy 60%	Cross-sectional design community (*n* = 19) nursing home (*n* = 30)	All having dementia	AQoL-8D (Australian general population)	Friendship Scale^c^	Community: 15.11 (3.63) U:0.43 (0.18)	Nursing Home: 10.80 (3.68) U:0.30 (0.20)	*Isolated/ low level of social support: *[12–15] 15.11-*,* U:0.43 *Very socially isolated: [0–11]* 10.80, U:0.30
Jansons et al., Australia [34]	≥ 65 years mean age (SD) *Home-based programme:* Mean age (SD): 66 (13) Women: 75% Married 61% *Gym-based programme* Mean age (SD): 68 (11) Women: 54% Married 78%	Interventional study in adults with chronic health conditions such as cancer or diabetes: Home-based programme with telephone support (*n* = 51) Gym-based exercise programme (*n* = 54)	Multiple co-morbidities; poor or declining mobility; physical de-conditioning; or a combination of these problems	EQ-5D-3L (UK general population)	Friendship Scale^c^	Home^d^ *Baseline*: 19.2 (3.9) U:0.67 (0.21) *3-months* 19.0 (4.4) U:0.65 (0.22) *6-months* 19.8 (4.1) U:0.67 (0.25) *9-months* 19.1 (4.5) U:0.66 90.22) *12-months* 17.1 (4.4) U:0.68 (0.22)	Gym *Baseline:* 19.2 (4.2) U:0.63 (0.26) *3-months* 19.2 (4.5) U:0.59 (0.28) *6-months* 19.7 (3.4) U:0.67 (0.25) *9-months* 20.0 (3.9) U:0.66 (0.23) *12-months* 17.5 (4.2) U:0.67 (0.25)	*Socially connected *^*d*^ [19–21] 19.2, U:0.67 19.0, U:0.65 19.8, U:0.67 19.1, U:0.66 19.2, U:0.63 19.2, U:0.59 19.7, U:0.67 20.0, U:0.66 *Some social support:* [16–18] 17.1, U:0.68 17.5, U:0.67
Hawton et al., United Kingdom [38]	Community living > 50 years of age, *at risk of social isolation (n* = *232):* mean age (SD): 72 (11.8) living alone: 49.6% *socially isolated (n* = *94):* mean age (SD): 69.7 (12.0) living alone: 38.3% *severely socially isolated (n* = *67):* mean age (SD): 69.8 (12.1) living alone: 50.7	Cross-Sectional Study (*n* = 393)	*At risk of social isolation* number of physical co-morbidities 2.0 (1.3) clinically depressed: 37.6% *socially isolated:* number of physical co-morbidities: 1.6 (1.3) clinically depressed: 34.4% *severely socially* number of physical co-morbidities 2.1 (1.3) clinically depressed: 65.2%	EQ-5D-3L SF-6D (UK general population)	Social Health Battery^e^			*At risk* EQ-5D: 0.65 SF-6D: 0.67 *Socially Isolated* EQ-5D: 0.69 SF-6D: 0.67 *Severely socially isolated* EQ-5D: 0.50 (0.32) SF-6D: 0.59 (0.12)
Gardner et al., Australia [33]	18–25 years old Mean age (SD): 21 (2.21) Women: 48% Living with family: 72%	Exploratory observational study (*n* = 159)	Serious mental illness^f^	AQoL-8D (Australian general population)	The Social Inclusion Scale^g^	Socially isolated: 13.41 (3.83) U:0.42 (0.21)		
Sarant et al., Australia [36]	Mean age (SD) 72 (6.8)	Interventional study investigating the impact of cochlear implants (*n* = 20)	with severe–profound hearing loss	HUI3 (Not stated)	The Lubben Social Network Scale-10 item revised ^h^	Baseline: 45.36, U:0.56 18 months: 44.77, U:0.67		

^a^The Lubben Social Network Scale- 6 item, the scores range between 0 and 30, with a higher score indicating more social engagement. Score less than 12 indicate being socially isolated

^b^Median and (IQR)

^c^Friendship Scale: A score of ‘0’ indicates complete social isolation and a score of ‘24’ indicates high social connectedness, categorised into 0–11 very socially isolated, 12–15 isolated or low level social support, 16–18 some social support, 19–21 socially connected, 22-24very socially connected

^d^This RCT collected loneliness and utility data across 5 measurement points (baseline, 3 months, 6 months, 9 months, 24 months) for the intervention and the control group

^e^An item from Rand Social Health Battery ‘How many times a year do you get together with friends and relatives, e.g. going out together or visiting each other’s homes?’. Two additional questions from the Social Health Battery addressed social activity: ‘How many close friends and family do you have?’ and ‘About how many clubs/groups/ organisations do you belong to?’. Six items from the Medical Outcomes Study Social Support Survey were used to measure the availability of aspects of functional social support

^f^Serious mental illness included depressive disorders, anxiety disorders, schizophrenia, personality disorder, trauma-related disorder, bipolar related disorder, eating disorder, obsessive–compulsive and related disorders, substance-related and addictive disorders

^g^The Social Inclusion Scale, a 19-item self-report measure of social inclusion. It contains a sub-scale of a Social Isolation (range 5–20, higher scores indicate more social isolation)

^h^The Lubben Social Network Scale-revised the score ranges between 0 and 50, with a higher score indicating more social engagement a score less than 20 indicate person with high risk of social isolation

### Studies measuring Loneliness

Studies that included a loneliness measure valued health states using various MAUIs, including the EQ-5D-3L [32, 37, 39, 41–43, 45–47, 49], AQoL-8D [33, 35], 15D [48], HUI2 [44] and HUI3 [36]. Measures of loneliness included the UCLA 20-item scale [32, 37, 46], R-UCLA 20-item [33], R-UCLA 3-item [35, 41], 11-item De Jong Gierveld Scale [36, 39, 42, 43], 6-item De Jong Gierveld Scale [47] and study specific questions asking individuals directly whether they are lonely or not [35, 44, 45, 48, 49].

**Fig. 2:**
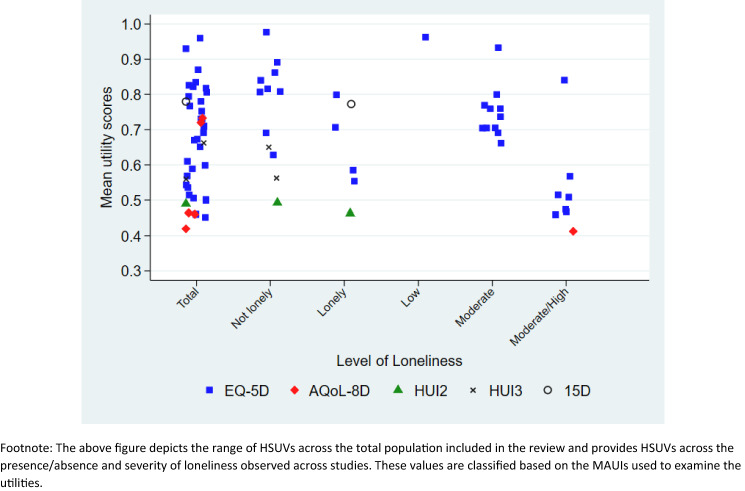
Reported Utility values across levels of loneliness based on the employed MAUIs

Figure 2 demonstrates the reported HSUVs using various MAUIs by the reported degree of loneliness. The HSUVs of the overall sample included in the studies ranged from as low as 0.42 to as high as 0.95. Those identified as ‘being lonely’ or ‘not lonely’ by the respective study or using the categorisation based on cut-off points of the loneliness-specific measures, reported HSUVs ranging from 0.46 to 0.81 for the ‘lonely’ sample, whereas scores ranged from 0.5 to 0.95 for those who reported ‘not being lonely’. Only one study reported HSUVs by the degree of loneliness using the EQ-5D-3L (HSUVs: low = 0.97, moderate = 0.93, moderately high = 0.86) [32]. This study demonstrated a significant difference among the HSUVs across the levels of loneliness. Remaining studies were categorised by levels of loneliness using cut-off points from Zhu et al. [32]. HSUVs for ‘low level of loneliness’ reported by one study was 0.97 [32], whereas HSUVs for ‘moderate level of loneliness’ and ‘moderately high’ ranged from 0.65 to 0.93 and from 0.42 to 0.86 [32], respectively. When using the AQoL-8D, HUI2 and HUI3, lower HSUVs were reported compared with scores using the EQ-5D-3L.

The included studies consisted of participants with varying population characteristics and health conditions. Two studies focused specifically on participants having a subjective feeling of loneliness, which reported a HSUV of 0.46 [43] and 0.78 [48]. Two studies, which included a population with serious mental health problems, such as depressive disorders, anxiety disorders, schizophrenia, personality disorder and others, reported HSUV of 0.81 associated with moderate levels of loneliness [46] and 0.42 with moderately high levels of loneliness [33]. While most studies had a mean age greater than 50 years, one study considered younger adults with serious mental illness and reported that high levels of loneliness were associated with a HSUV of 0.42 [33]. A study which explored HSUVs among people with chronic diseases reported ‘moderately lonely’ scores with HSUVs ranging from 0.62 to 0.72 [42]. Two studies, including participants with chronic health conditions, reported HSUVs for those ‘feeling lonely’ of 0.46, using the HUI2 [44] and 0.56 using EQ-5D-3L [49] which were lower than the HSUVs of those ‘not feeling lonely’ 0.50 [44] and 0.60 [49]). Two studies explored HSUVs in individuals with sight loss [37], HIV [45], skin disease [41] and severe–profound hearing loss [36], with HSUVs ranging from 0.51 to 0.89 using the HUI3 and EQ-5D-5L.

A study by Rodriguez-Blazquez et al. explored well-being among institutionalised and non-institutionalised older adults, showing that both groups were ‘feeling lonely’, with those institutionalised having a greater loneliness score as well as a lower HSUVs of 0.57 vs 0.81 in those non-institutionalised measured by the EQ-5D-3L [47]. There was one study that did not report any health conditions in study participants that were classified as ‘moderately lonely’ with HSUVs ranging from 0.71 to 0.77, using EQ-5D-3L [39].

Ideally, to generate more precise estimates of the HSUVs of lonely/socially isolated individuals, HSUVs could be pooled together using meta-analysis, thereby providing an average effect size. However, given the limited number of studies, differences in the utility valuation techniques, scoring algorithms, country tariffs and health conditions across study populations, which all lead to high heterogeneity, a quantitative meta-analysis was not possible [51].

### Studies measuring social isolation

Table 4 describes the study characteristics and reported utility weights of six studies measuring social isolation. HSUVs were measured using the EQ-5D-5L [40, 41], EQ-5D-3L [34, 38], AQoL-8D [33, 50], SF-6D [38] and HUI3 [36]. The various instruments used to measure social isolation included the Lubben Social Network scale (LSNS) 6-item [40, 41], Revised-Lubben Social Network scale [36], Friendship scale [34, 50], Social Health Battery [38] and Social inclusion scale [33]. HSUVs by levels of social isolation could not be reported as these measures were not consistently reported across studies.

HSUVs for those reported being socially isolated ranged from 0.3 [50] to 0.87 [40]. A study which explored severity of social isolation in participants with physical co-morbidities as well as depression, reported that those who were severely socially isolated had lower HSUVs of 0.50 and 0.59 measured by the EQ-5D-3L and SF-6D, respectively, compared to the HSUVs of 0.65 and 0.67 in those who reported to be at risk of social isolation [38]. Two other studies included participants with physical co-morbidities [34, 40]. The first study reported HSUVs ranging from 0.63 to 0.87 in those who were socially isolated and 0.81 to 0.94 in those who were not socially isolated [40]. In the second study, the participants reported being socially connected with HSUVs ranging from 0.59 to 0.68 [34]. One study assessed social isolation in participants aged 60 years and above experiencing dementia based on their living arrangements. The participants living in nursing homes reported to be ‘very socially isolated’ with HSUV of 0.30 which is lower when compared to the participants living in a community setting reporting to be ‘isolated’ with HSUV of 0.43 [50]. This review also included an article, which explored social isolation in younger adults with serious mental illness reporting a HSUV of 0.43 [33].

## Discussion

The aim of this study was to provide an overview of the current state of evidence for the impact of loneliness and/or social isolation on HSUVs across all age groups, establishing a better understanding of the associated disease burden. Compared to the UK EQ-5D population norms of 0.852 [52] and the Australian AQoL-8D population norms of 0.81 [53], HSUVs associated with loneliness and/or social isolation identified by this review were lower. This suggests that participants experiencing loneliness and/or social isolation have lower quality of life compared to the general population. Moreover, the lowest HSUV reported for those with a serious mental illness experiencing loneliness and social isolation was 0.42 as measured by the AQoL-8D [33], which is even lower compared to the mean HSUV of 0.67 associated with high prevalence mental disorders in Australia reported using the AQoL-4D [54]. However, these low values may not reflect the true burden of loneliness and social isolation, as HSUVs may have been influenced by many underlying factors, such as the health conditions of the participants, variations in the methods used for the assessment of HSUVs as well as the measurement of loneliness and/ or social isolation. Therefore, the derived HSUVs need to be interpreted carefully, considering many confounding factors that are outlined in the following discussion.

### Different measurement tools used to assess loneliness/social isolation

The UCLA loneliness scale and the De Jong Gierveld loneliness scale were the most frequently used measures for assessing loneliness, whereas the Lubben Social Network scale and Friendship scale were most often used for measuring social isolation. However, we found variations in the use of terminology and concepts of loneliness and social isolation and the corresponding use of measures. For instance, a study that claimed to measure social isolation used the R-UCLA, which is a measure of subjective loneliness [35]. The De Jong Gierveld Loneliness scale differentiates between two types of loneliness, including social loneliness and emotional loneliness. Only one of the 5 studies which used this instrument to measure loneliness reported scores within the two dimensions [39]. As our review focused only on overall loneliness scores, further studies could explore HSUVs associated with both types, social and emotional loneliness. Some included studies categorised levels of social isolation referring to concepts such as ‘social support’ or ‘social connectedness’ [34, 50], which may not be reliable indicators of social isolation [55] demonstrating lack of conceptual clarity around these terms. While the above stated instruments are validated measures [56–59], we also found many studies that used study specific single item questions to identify people experiencing loneliness. While it is problematic to use non-validated measures, it is also known that such direct measurement techniques, generally result in under-reporting of loneliness due to the associated social stigma attached to loneliness [60].

### Difference across levels of loneliness/social isolation

Among those studies which included participants with a subjective feeling of loneliness [32, 33, 48, 49], one study reported HSUVs by the degree of loneliness [32]. This study reported decreasing HSUVs by increasing levels of loneliness as measured by the EQ-5D-3L. Two studies by Taube et al. and Maxwell et al. reported HSUVs of those lonely vs no lonely and found that the HSUVs were lower in the lonely group than those not feeling lonely [44, 49]. Another study, which categorised HSUVs based on levels of social isolation, reported lower HSUVs in the severely socially isolated group compared to those at a lower risk of social isolation [38]. Similar observations were noticed in a study by Nikmat et al. which reported a lower HSUV in those who were very socially isolated compared to those who had lower levels of social support. [50]. However, in all the above studies, the participants reported co-morbidities, which makes it challenging to extract the excess health burden associated with loneliness.

While the focus of this review was on both loneliness and social isolation, it remains unclear whether there is a greater health burden, in terms of reductions in HSUV, associated with loneliness and/or social isolation. Gardner et al. and Sarant et al. were the only two studies that included measures of both loneliness and social isolation [33, 36], although the later study sample neither experienced loneliness nor social isolation. Participants in the Gardner et al. study consisting of young adults with serious mental illness, reported higher levels of loneliness while not being socially isolated with a HSUV of 0.42. As this study did not adjust for health conditions, whether the lower HSUV reflects the burden of loneliness cannot be clearly confirmed. Although all studies have included participants with pre-existing health conditions, it would be of interest to further explore HSUVs in those experiencing loneliness/social isolation without any underlying health conditions.

### Different MAUIs used across studies

It has been previously shown that different MAUIs produce different HSUVs [61] and it was observed that there were several MAUIs used to assess HSUVs across the included studies. The impact of the choice of MAUI was observed in the study by Hawton et al. (2010), where different HSUVs across different levels of social isolation were reported when using the EQ-5D-3L compared with the SF-6D [38]. These differences are likely due to the conceptual differences between MAUIs. For example, the EQ-5D-3L, used in most of the studies to derive HSUVs, mainly focuses on physical health [62] but does not contain any items related to social functioning. Contrastingly, the SF-6D contains equal item in both physical and psychosocial domains. Similarly, the AQoL-8D includes items related to social isolation, social exclusion and satisfaction with close relationships, which could potentially capture the feeling of loneliness/social isolation. In our review, the lowest HSUV of 0.42 for participants classified as moderately lonely’ [33] and the lowest HSUV of 0.30 for those reporting as being highly socially isolated [50], was measured using the AQoL-8D. This suggests that the AQoL-8D may be more sensitive in capturing aspects of loneliness/social isolation. However, further research is needed to validate this observation as the studies included patient with serious mental illness [33] and chronic conditions [35] and thus, it cannot be ruled out whether decrements in HSUVs were solely driven by loneliness.

### Presence of multi-morbidities

Most of the studies included older adults with various physical and mental health conditions, which means that the HSUVs observed could have been driven by the presence of existing multi-morbidities. Previous studies have reported reductions in HSUVs associated with health conditions such as serious mental illness [61]. As loneliness and social isolation have negative impacts on the physical and psychological health of older people leading to serious health consequences [63], it is difficult to understand the excess burden attributable to loneliness and/or social isolation. There were studies that included specific health conditions, such as sight loss [37], diabetes [35], serious mental illness [33] and profound hearing loss [36]. Therefore, to inform future modelling studies, there will be a need for estimates of the joined health condition HSUV, considering both co-morbidities and levels of loneliness [64].

### Difference among community-dwelling and institutionalised populations

The living situation of study participants could also affect HSUVs reported in this review. Only two studies compared HSVUs between older people living in residential aged care and community settings [47, 50]. Both studies reported a lower HSUV in those institutionalised, who also reported higher levels of loneliness [47] and social isolation [50]. This is consistent with previous literature, which has stated that loneliness is higher in those living in institutions compared to community-dwelling older adults [56, 65]. However, it is important to consider the various confounding aspects to extract out the true burden on loneliness and/or social isolation.

### Limitations and further research

The current paucity of published utility weights by severity status of loneliness and social isolation is reflected in the lack of cost-utility analysis of loneliness interventions. This is the first systematic review that has explored HSUVs associated with loneliness and social isolation using a broad range of inclusion criteria. The HSUVs identified could serve as input parameters for future modelled economic evaluations, although the uncertainty within the body of literature needs to be considered for appropriate use of HSUVs. Our study has certain limitations and results should be interpreted with caution. First, we used cut-off points reported by Zhu et al. to define levels of loneliness [32]. It is possible that different categorisation of cut-off points may have resulted in different corresponding HSUVs. Second, due to heterogeneity of the studies, we could not conduct a meta-analysis to synthesise estimates of health utilities [66]. Third, a small number of studies were included in this review, which underscores the existing research gap and the need for future studies. Due to lack of standardised checklists for assessing the quality of HSUVs, we did not conduct quality assessments of the included studies. The arrival of the Covid-19 pandemic and its impact on the burden of loneliness and social isolation has been a growing concern. Although we re-ran the search at the beginning of 2021, no studies exploring the impact of Covid-19 had been published which met the criteria for inclusion in the review. We may expect more studies to arise, incorporating the additional burden on loneliness and social isolation due to the Covid-19 pandemic.

## Conclusion

This study is the first systematic review providing summary of existing HSUVs by severity of loneliness and/or social isolation across age groups, demonstrating the paucity of evidence available around specific health burden of loneliness and/or social isolation. With a broad range of reported HSUVs, this review highlights the challenges in identifying reliable estimates of HSUVs that are essential input parameters in model-based economic evaluations of interventions targeted to address loneliness and /or social isolation. These challenges mainly arise due to (i) differences in the scoring algorithms and descriptive systems of MAUIs that result in different weights in deriving the HSUVs, (ii) differences in the measurement of loneliness and social isolation, (iii) and the confounding effects of health conditions of the respondents and different settings. Despite these observed challenges, this review reflects detriments in utility which are important indicators of the burden associated with loneliness and social isolation. Further studies exploring HSUVs across levels of loneliness and social isolation in a systematic and justified method are encouraged.

## Supplementary Information

Below is the link to the electronic supplementary material.

Supplementary file1 (DOCX 12 kb)
