# Supplementary material for: The impact of loneliness and social isolation on health state utility values: a systematic literature review
Source: Qual Life Res. 2022 Jan 24;31(7):1977–97. doi: 10.1007/s11136-021-03063-1 (PMC8785005; doi:10.1007/s11136-021-03063-1)
Supplement: Supplementary file 1 — Supplementary file1 (DOCX 12 kb) [file 11136_2021_3063_MOESM1_ESM.docx]

Appendix 1:

**Search terms/concepts:**

| Concept 1 | Loneliness and/or Social Isolation | lonel* OR “Solitude” OR “social* isolat*” OR "Social* exclu*" OR “social* distan*” OR “social environment” |
| --- | --- | --- |
| Concept 2 | Measures of loneliness | “UCLA” OR “De Jong Gierveld” |
| MESH terms |  | Loneliness  Social Isolation |
| Concept 3 | Health Utilities | “health state utilit*” OR “health utilit*” OR “utilit* OR “quality of life” OR “wellbeing” OR “health state valu*” OR “health value*” OR “health status” OR “QOL” OR “multi attribute utility instrument*” OR “multi-attribute utilit*” OR “quality adjusted life year*” OR “QALY*” OR “Short form 36” OR “Short form 12” OR “SF-36” OR “SF-12” OR “HUI*” OR “Aqol*” OR “15D” OR “time trade off” OR “standard gamble” OR “visual analogue scale” OR “VAS scale” OR “direct elicitation” OR “indirect elicitation” OR “EurolQoL*” OR “EQ-5D*” OR “economic evaluation*” OR “cost utilit*” OR “cost effectiv*” OR “economic model*” OR “return on investment*” OR “value for money” OR “cost consequence*” OR “cost saving*” OR “ROI” |
| MESH term |  | Quality-adjusted life years/ |
| Combine 1 |  | Concept 1 OR Concept 2 |
| Combine 2 |  | Combine 1 AND Concept 3 |
